# Preoperative Factors Associated with Surgical Complexity and Postoperative Outcomes in Patients Undergoing Robotic Anatomical Segmentectomy

**DOI:** 10.1093/icvts/ivaf294

**Published:** 2025-12-04

**Authors:** Oscar Colmenares, M Teresa Gómez-Hernández, Cristina E Rivas, Marta G Fuentes, Mario Manama, Francisco Gómez, Carmen Taboada, Clara Forcada, María Caro, Marcelo F Jiménez

**Affiliations:** Service of Thoracic Surgery, Salamanca University Hospital, Salamanca, 37007, Spain; Salamanca Institute of Biomedical Research, Salamanca, 37007, Spain; Service of Thoracic Surgery, Salamanca University Hospital, Salamanca, 37007, Spain; Salamanca Institute of Biomedical Research, Salamanca, 37007, Spain; University of Salamanca, Salamanca, 37007, Spain; Service of Thoracic Surgery, Salamanca University Hospital, Salamanca, 37007, Spain; Salamanca Institute of Biomedical Research, Salamanca, 37007, Spain; University of Salamanca, Salamanca, 37007, Spain; Service of Thoracic Surgery, Salamanca University Hospital, Salamanca, 37007, Spain; Salamanca Institute of Biomedical Research, Salamanca, 37007, Spain; University of Salamanca, Salamanca, 37007, Spain; Service of Thoracic Surgery, Salamanca University Hospital, Salamanca, 37007, Spain; Salamanca Institute of Biomedical Research, Salamanca, 37007, Spain; Service of Thoracic Surgery, Salamanca University Hospital, Salamanca, 37007, Spain; Service of Thoracic Surgery, Salamanca University Hospital, Salamanca, 37007, Spain; Service of Thoracic Surgery, Salamanca University Hospital, Salamanca, 37007, Spain; Salamanca Institute of Biomedical Research, Salamanca, 37007, Spain; University of Salamanca, Salamanca, 37007, Spain; Service of Thoracic Surgery, Salamanca University Hospital, Salamanca, 37007, Spain; Service of Thoracic Surgery, Salamanca University Hospital, Salamanca, 37007, Spain; Salamanca Institute of Biomedical Research, Salamanca, 37007, Spain; University of Salamanca, Salamanca, 37007, Spain

**Keywords:** segmentectomy, complexity, RATS, early-outcomes

## Abstract

**Objectives:**

Anatomical segmentectomy is increasingly used for early-stage lung cancer due to its parenchymal-sparing benefits. However, it remains technically challenging, and procedural complexity is often inconsistently defined. Robotic-assisted surgery, offering enhanced dexterity and visualization, has become more widespread but still requires high technical expertise. This study aimed to identify preoperative factors associated with procedural complexity and evaluate its impact on postoperative outcomes.

**Methods:**

This single-centre cohort study included 160 consecutive patients who underwent robotic segmentectomy by 2 expert surgeons between November 2018 and August 2025. Complex procedures were defined as those with operative time >125 min (75th percentile), conversion to another surgical approach, or changes in the planned resection due to intraoperative technical challenges. Logistic regression was used to identify preoperative variables associated with complexity. Postoperative outcomes were compared between complex and non-complex cases.

**Results:**

Thirty-seven segmentectomies (23.1%) were classified as complex. Predictors of complexity included age (odds ratio [OR] = 1.042, *P* = .063), transverse pleural diameter (OR = 0.716, *P* = .089), and number of staple planes (OR = 1.644, *P* = .058), while the presence of emphysema (OR = 0.428, *P* = .076) appeared to be protective. Mortality, overall morbidity, prolonged air leak, and readmission rates were similar between groups. However, complex cases had significantly higher rates of major morbidity (13.5% vs 1.6%, *P* = .008), reintervention (10.8% vs 0.8%, *P* = .010), and longer hospital stays (median 3 vs 2 days, *P* = .004).

**Conclusions:**

This exploratory analysis identified preoperative factors associated with procedural complexity in robotic segmentectomy. These findings may help improve patient selection, surgical planning, resource allocation, and structured training.

## Graphical abstract

**Figure:**
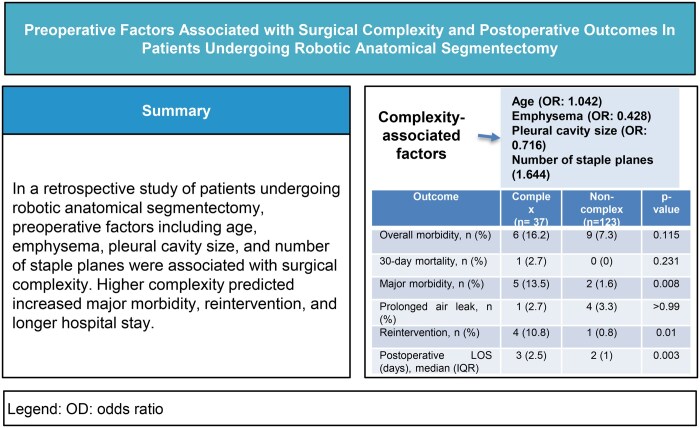

## INTRODUCTION

Pulmonary anatomical segmentectomy has become increasingly relevant in thoracic surgery. Recent data from the European Society of Thoracic Surgeons (ESTS) database indicate that the frequency of these procedures has risen markedly, now accounting for 10% of all lung resections across a range of diagnoses.^1^

This growing adoption is driven by evidence from recent trials (JCOG0802/WJOG4607L, JCOG1211, and CALGB 140503), which support segmentectomy as an oncological valid alternative to lobectomy for small, early-stage non-small cell lung cancer (NSCLC) without nodal involvement.^2–4^ The widespread detection of small nodules and ground-glass opacities through computed tomography (CT) screening is also contributing to its increasing use.^5^ Segmentectomy is further indicated in patients with limited cardiopulmonary reserve, poor performance status, or advanced age^6^ and is routinely performed for metastatic tumours and select benign conditions such as inflammatory lesions, infections, or malformations.^7^

Anatomical segmentectomy is technically demanding due to the need for precise dissection of segmental structures. While traditionally performed via thoracotomy or video-assisted thoracoscopic surgery (VATS), robot-assisted thoracoscopic surgery (RATS) has recently emerged as a promising minimally invasive alternative, offering enhanced vision, ergonomics, and instrument control.^8^^,^^9^ However, despite its technical advantages, robotic segmentectomy remains a highly complex procedure that demands advanced anatomical knowledge and surgical expertise, which continues to hinder its broader adoption in routine clinical practice.

To date, the definition of segmentectomy complexity remains inconsistent. Although complexity is often linked to the need for multiplanar dissection of intersegmental planes,^10^ other anatomical and patient-related factors may influence surgical difficulty. An objective tool to quantify procedural complexity could guide stepwise surgical training, help select appropriate cases for competence assessment, and optimize operating room scheduling by matching case difficulty with surgeon expertise—ultimately enhancing team efficiency and patient safety.

The objective of this study was to identify preoperative factors—based on patient-related characteristics and CT findings—associated with increased procedural complexity in robotic segmentectomy. Recognizing these factors may help to support preoperative planning, optimize operating room scheduling, and facilitate surgical training during the early stages of the learning curve.

## METHODS

### Ethical statement

The need for Clinical Research Ethics Committee approval for this project was waived according to our institutional regulations because the study was a retrospective cohort one based solely on anonymized patient data collected from routine clinical practice. Likewise, the need for individual patient consent was waived because no identifiable personal information was used, and the study involved no intervention or contact with patients.

### Study design, data source, and patients

This is a retrospective analysis on 160 consecutive RATS segmentectomy patients operated between June 2018 and August 2025 by 2 Intuitive Surgical-certified robotic surgeons with a robotic experience of > 200 robotic procedures. No formal power calculation was performed due to the exploratory nature of the study. All data were obtained from an institutional prospective, computerized database. Quality control of the data was assured by 2 successive audits made by the quality control manager of the unit, the first one at the moment of patient hospital discharge and the second before uploading the final records in the hospital central archive. The database included clinical, radiological, and pathological variables, type of resection, and intraoperative and postoperative data. Definition of variables was based on the standardized document of the Society of Thoracic Surgeons and the European Society of Thoracic Surgeons (ESTS)^11^ and the classification of surgical complications proposed by Dindo et al.^12^

### Operative technique

Preoperative planning was conducted for all cases using dedicated software (Laia XR, Arsoft, Salamanca, Spain) capable of displaying 3-dimensional reconstructions of the pulmonary anatomy. The robotic programme was initiated at our institution in May 2018. Segmentectomies began to be performed via this approach after the initial learning curve was surpassed, approximately 6 months later.^13^ The operative technique for robotic anatomical lung resection was standardized and consistently applied, as previously described.^14^ Basically, patients were positioned in lateral decubitus with slight hip flexion. A completely portal robotic approach using 4 arms was employed. An 8-mm robotic camera port was placed in the eighth intercostal space (ICS) at the mid-axillary line, and the thoracic cavity was explored using a 0° endoscopic camera. Two additional 8-mm robotic trocars were inserted in the eighth ICS—one at the auscultatory triangle and the other at the scapular line. A 12-mm robotic port was positioned in the seventh ICS at the anterior axillary line, near the level of the diaphragm. An auxiliary 10-mm port was introduced in the ninth ICS, between the camera port and the anterior robotic port, creating a triangulated configuration. CO_2_ insufflation was applied at a pressure of 6-10 mmHg.

The segmentectomy was performed by first dissecting the fissure and systematically removing the lymph nodes surrounding the segmental artery and bronchus. The segmental arteries were individually dissected and divided using a stapler. Segmental veins were usually divided together with the parenchyma, unless their individual dissection at the segmental hilum was required to expose adjacent structures. The segmental bronchus was then isolated, and its correct identification was confirmed using a combination of intraoperative bronchoscopy and near-infrared fluorescence imaging, as previously described.^15^ The bronchus was subsequently transected using a stapler. In all cases, the intersegmental plane was identified after intravenous injection of indocyanine green (ICG) and visualized with the Firefly fluorescence imaging system integrated into the robotic platform. The intersegmental plane was then divided with sequential stapler firings, taking care to preserve the intersegmental veins. In general, robotic endoscopic staplers were used for vascular and bronchial structures, whereas motorized staplers were employed for parenchymal division through the assistant port. The specimen was retrieved by slightly enlarging the anterior port.

### Statistical analysis

Complex operations were defined as those meeting any of the following criteria: (1) operative time exceeding the 75th percentile within the study cohort; (2) intraoperative conversion to VATS or thoracotomy; or (3) a change in the planned extent of resection due to intraoperative technical difficulties.

The following clinical and surgical variables were evaluated for potential association with surgical complexity: age, sex, body mass index (BMI), forced expiratory volume in 1 second (FEV1%), diffusing capacity of the lung for carbon monoxide (DLCO%), history of coronary artery disease, cerebrovascular disease, diabetes mellitus, previous ipsilateral thoracic surgery, side of the operation (right or left), and the stapling technique used to define the intersegmental plane (linear, V-shaped, or 3-dimensional).^16^ Linear stapling involves a single, mostly straight intersegmental plane (eg, S6, basal, lingular, or left superior segments). If the resection extends into an adjacent subsegment (eg, left S1 + 2), the plane is slightly curved but can be easily stapled. V- or U-shaped stapling includes 2 or 3 planes forming a V- or U-shape (eg, right S1, left S3). Three-dimensional stapling involves intersegmental planes forming a cuboid-shaped segment, often influenced by diaphragmatic phasing (eg, S7, S8, S9). In addition, specific chest CT findings were analysed, including fissure completeness (complete vs incomplete), radiological evidence of pleural adhesions, presence of emphysema, visibility of hilar or interlobar lymph nodes on CT scan, transverse diameter of the pleural space (measured from the rib cage to the tracheal carina), and longitudinal diameter (from the apex to the diaphragmatic cupula).

A rule of thumb was applied assuming that at least 10 patients per covariate are required to ensure adequate model stability, which allowed the analysis to explore up to 10-15 covariates.

Potential predictors of complex segmentectomy were first explored through univariable logistic regression. Variables with a *P*-value < .20 were eligible for multivariable logistic regression using stepwise backward selection (Wald). A retention threshold of *P* < .10 was chosen given the small sample size and the exploratory nature of the study. Multicollinearity was assessed using tolerance and variance inflation factor (VIF) values.

This multivariable analysis was intended as an exploratory approach to identify preoperative factors associated with procedural complexity, rather than to develop a prognostic model. Model discrimination was assessed graphically using the area under the receiver operating characteristic (ROC) curve, and model calibration was evaluated with the Hosmer-Lemeshow goodness-of-fit test.

Finally, patients undergoing complex versus non-complex segmentectomy were compared using Fisher’s exact test or Pearson’s chi-squared test for categorical variables (Chi-square tests were applied when all expected cell counts were ≥5, and Fisher’s exact test was used when any expected count was <5), and the Mann-Whitney *U* test for continuous variables. A 2-tailed *P*-value <.05 was considered statistically significant.

These criteria were pre-specified in the statistical analysis plan. All statistical analyses were performed using SPSS Statistics, version 26 (IBM Corp., Armonk, NY, USA).

The manuscript adheres to the STROBE (Strengthening the Reporting of Observational Studies in Epidemiology) guidelines.

## RESULTS

A total of 160 patients were included in the study. The majority (70%) had a final pathological diagnosis of primary lung cancer, 9.4% presented with pulmonary metastases, and the remaining cases corresponded to other histopathological entities. The median operative time was 115 min (interquartile range [IQR]: 90-125), with 36 procedures (22.5%) exceeding the upper quartile (>125 min). Conversion to thoracotomy was required in 3 cases (1.88%) due to oncological concerns (*n* = 1), technical difficulties (*n* = 1), or intraoperative bleeding (*n* = 1). In 3 additional patients (1.88%), the resection ultimately performed differed from the preoperative plan due to inadvertent section of a pulmonary vein (*n* = 1), absence of the targeted nodule in the *ex vivo* specimen (*n* = 1), or a residual segment that was considered too small to preserve (*n* = 1). Therefore, 37 robotic segmentectomies were classified as complex (23.1%). The exact segmental location of the procedures is presented in **Table 1**. **Table 2** provides an overview of the clinical, radiological, and surgical characteristics of the patients, along with the results of the univariable analysis.

**Table 1.** Segment Location During Complex and Non-complex Segmentectomies

Segment/s, *n* (%)	Complex	Non-complex
	(*n* = 37)	(*n* = 123)
S1	3 (8.1)	17 (13.8)
S1 + 2	1 (2.7)	12 (9.8)
S1 + 2+3	0 (0)	1 (0.8)
S2	3 (8.1)	18 (14.6)
S2 + 6	0 (0)	3 (2.4)
S3	6 (16.2)	8 (6.5)
S4 + 5	3 (8.1)	7 (5.7)
S6	5 (13.5)	36 (29.3)
S6 + 10	1 (2.7)	0 (0)
S7	0 (0)	1 (0.8)
S7 + 8	0 (0)	3 (2.4)
S7 + 8+9 + 10	3 (8.1)	1 (0.8)
S8	2 (5.4)	8 (6.5)
S8 + 9+10	4 (10.8)	5 (4.1)
S9 + 10	6 (16.2)	3 (2.4)

**Table 2.** Univariable Analysis of Patient Characteristics and Surgical Features for the Outcome Complex Segmentectomy

Variable	Complex	Non-complex	OR	*P*-value
	(*n* = 37)	(*n* = 123)		
*Patients’ characteristics*				
Age, median (IQR)	70.09 (10.93)	67.56 (10.5)	1.034	.131
Male gender, *n* (%)	17 (45.9)	73 (59.3)	0.582	.152
BMI, median (IQR)	22.99 (4.95)	22.75 (7.3)	1.008	.843
FEV1, median (IQR)	96 (20)	96 (32)	1.001	.921
DLCO, median (IQR)	80 (22)	88 (26)	0.991	.327
Coronary disease, *n* (%)	2 (5.4)	5 (4.1)	1.349	.728
Cerebrovascular disease, *n* (%)	1 (2.7)	3 (2.4)	1.111	.928
Diabetes mellitus, *n* (%)	8 (21.6)	16 (13)	1.845	.203
Previous ipsilateral surgery, *n* (%)	1 (2.7)	5 (4.1)	0.656	.704
Right side, *n* (%)	19 (51.4)	75 (61)	0.676	.299
Tumoral size (mm), median (IQR)	13 (8.5)	15 (7)	0.961	.232
Stapling technique used to define the intersegmental plane, *n* (%)^a^			1.412	.155
- Linear	20 (54.1)	76 (61.8)		
- V-shaped	8 (21.6)	32 (26)		
- Three-dimensional	9 (24.3)	15 (12.2)		
*Chest CT findings*				
Incomplete fissure, *n* (%)	8 (21.6)	23 (18.7)	1.199	.694
Pleural adhesions, *n* (%)	6 (16.2)	22 (17.9)	1.125	.815
Emphysema, *n* (%)	7 (18.9)	42 (34.1)	0.450	.083
Presence of hilar or interlobar lymph nodes, *n* (%)	1 (2.7)	17 (13.8)	0.173	.094
Transverse diameter of the pleural space (cm), median (IQR)	11.5 (1.6)	11.9 (1.7)	0.718	.073
Longitudinal diameter of the pleural space (cm), median (IQR)	19.5 (3.8)	19.7 (3.5)	0.913	.232

a Stapling technique: linear stapling involves a single, mostly straight intersegmental plane. V- or U-shaped stapling includes 2 or 3 planes forming a V- or U-shape. Three-dimensional stapling involves intersegmental planes forming a cuboid-shaped segment.

Abbreviations: BMI, body mass index; CT, computed tomography; DLCO, carbon monoxide lung diffusion capacity; FEV1, forced expiratory volume in 1 second; IQR, interquartile range; OR, odds ratio.

**Table 3** presents the final model, which identified the following independent predictors of surgical complexity: age (odds ratio [OR] = 1.042, *P* = .063), transverse diameter of the pleural space (OR = 0.716, *P* = .089), and the stapling technique used to define the intersegmental plane (OR = 1.644, *P* = .058), while the presence of emphysema appeared to be protective (OR = 0.428, *P* = .076). Although none of the associations reached conventional statistical significance (*P* < .05), a retention threshold of *P* < .10 was applied to include clinically relevant variables and the final model comprised all factors potentially associated with surgical complexity. The prognostic model showed modest discriminatory ability (Area Under the Curve (AUC) = 0.687, 95% CI: 0.585-0.789) and acceptable calibration (Hosmer-Lemeshow *P* = .715).

**Table 3.** Multivariable Analysis of Risk Factors of Complex Segmentectomy

Variable	Adjusted odds ratio (95% CI)	*P*-value
Age	1.042 (0.998-1.089)	.063
Emphysema	0.428 (0.168-1.092)	.076
Transverse diameter of the pleural space	0.716 (0.487-1.052)	.089
Number of staple planes	1.644 (0.984-2.747)	.058

Abbreviation: 95% CI, 95% confidence interval.

### Perioperative outcomes complex versus non-complex cases

Comparison between complex and non-complex segmentectomies revealed no significant differences in overall mortality (2.7% vs 0%, *P* = .231) or overall morbidity (16.2% vs 7.3%, *P* = .115), including prolonged air leak (2.7% vs 3.3%, *P* > .999). In contrast, complex segmentectomies were associated with significantly higher rates of major morbidity (13.5% vs 1.6%, *P* = .008), reintervention (10.8% vs 0.8%, *P* = .010), and longer postoperative hospital stays (median: 3 vs 2 days, *P* = .004). Readmission rates were also similar between groups (2.7% and 0.8%, respectively; *P* = .410). Postoperative outcomes for both groups are summarized in **Table 4**.

**Table 4.** Postoperative Outcomes for Complex and Non-complex Segmentectomies

Outcome	Complex	Non-complex	*P*-value
	(*n* = 37)	(*n* = 123)	
Overall morbidity, *n* (%)	6 (16.2)	9 (7.3)	.115*
30-Day mortality, *n* (%)	1 (2.7)	0 (0)	.231*
Major morbidity, *n* (%)	5 (13.5)	2 (1.6)	.008*
Prolonged air leak, *n* (%)	1 (2.7)	4 (3.3)	>.99**
Reintervention, *n* (%)	4 (10.8)	1 (0.8)	.01*
Postoperative LOS (days), median (IQR)	3 (2.5)	2 (1)	.003**
Readmission, *n* (%)	1 (2.7)	1 (0.8)	.410*

* *P*-value Fisher’s exact test.

** *P*-value for Mann-Whitney *U*-test.

Abbreviations: IQR, interquartile range; LOS, length of hospital stay.

## DISCUSSION

Although segmentectomy has proven effective in early-stage lung cancer^17^^,^^18^ and its use has increased in recent years, its widespread adoption remains limited and variable, often depending on provider discretion. The robotic approach offers potential advantages that may improve segmentectomy outcomes. However, several barriers may contribute to this slow adoption, including the procedure’s technical demands, limited access to structured training, and logistical or financial constraints.

Segmentectomy has traditionally been classified as simple or complex based on anatomical criteria, particularly the configuration of the intersegmental plane. Simple segmentectomies—such as resection of the S6, upper division, or lingula—typically involve a single linear plane and are technically more straightforward. In contrast, complex segmentectomies—like those involving the S3, S9, or combined resections (eg, S1 + 3 or S9 + 10)—require dissection along multiple, non-linear planes and are more technically demanding.^19^ However, this anatomical classification may be somewhat arbitrary, as it overlooks clinical and demographic factors that also impact procedural complexity. To address this limitation—and following the example of Miyazaki et al,^20^ who developed an aggregate score to stratify the technical complexity of VATS lobectomy—we aimed to identify prognostic factors for complexity of robotic segmentectomy, incorporating both patient-related characteristics and preoperative imaging data. Such a tool may help identify patients at higher risk of a technically demanding procedure and support a more structured training pathway, particularly during mentorship or proctorship, ultimately enhancing patient safety and enabling more objective assessment of surgical competence.

An important strength of this study is the use of a composite definition of surgical complexity. We included 3 objective criteria: prolonged operative time, conversion to open surgery, and an unplanned change in the extent of resection due to intraoperative technical difficulties. Each component has strong face validity. Operative time has been recently associated with increased postoperative morbidity after VATS lobectomy^21^ and represents a logical surrogate for procedural complexity. The conversion rate in our cohort (1.88%) aligns with previously reported data^22^^,^^23^ and also reflects intraoperative difficulty. Finally, deviations from the planned resection—despite systematic preoperative planning—due to factors such as misinterpretation of the broncho vascular anatomy, serve as additional indicators of technical challenge. We acknowledge, however, that the definition of complexity inevitably involves a certain degree of arbitrariness, as no standardized or universally accepted classification currently exists. Our composite definition was designed to capture real intraoperative challenges beyond purely anatomical considerations, providing a more pragmatic and clinically meaningful perspective.

We identified 3 independent factors potentially associated with complex operations—age, pleural cavity size, and the stapling technique used to complete the intersegmental plane—as well as 1 factor that appeared to be protective: the presence of emphysema. All of these maintained independent associations with complexity in multivariable regression analysis. Each risk factor likely influences technical complexity through different mechanisms, mostly related to challenges in surgical dissection. However, given that none of the associations reached conventional statistical significance (*P* < .05), the results should be interpreted with caution.

Advanced age may be associated with increased chronic inflammation, fibrotic changes, and tissue frailty, all of which can complicate dissection and increase the technical difficulty of the procedure. Pleural cavity size also had a relevant impact, particularly in cases with limited intrathoracic space, where instrument manoeuvrability is reduced and the recommended minimum 7 cm distance between ports cannot always be achieved. As anticipated, the stapling technique used to complete the intersegmental division was the most critical determinant of complexity, reflecting the geometric intricacy of the segmental anatomy and the technical demands of accurately completing parenchymal resection.^16^

Interestingly, in our series, emphysema appeared as a protective factor against procedural complexity. Although such cases are often considered technically demanding due to parenchymal fragility or chronic airway disease, we hypothesize that bronchovascular dissection may be facilitated by the softer consistency of emphysematous lung tissue and reduced adherence to bronchovascular structures. Nevertheless, this observation should be interpreted cautiously, as emphysema is known to increase the risk of postoperative complications such as prolonged air leak. However, no differences in prolonged air leak rates were observed between complex and non-complex procedures in our cohort. Our composite definition of complexity was intentionally restricted to intraoperative parameters, which might account for this apparent protective effect.

When comparing operative outcomes between complex and simple segmentectomies in our series, we found no differences in mortality or overall morbidity, including prolonged air leak. These results are consistent with previous reports.^22^^,^^24–27^ Likewise, Bertolaccini et al^28^ recently published a systematic review and meta-analysis demonstrating that simple and complex segmentectomies yield comparable postoperative outcomes—particularly in terms of complication rates and length of hospital stay—despite longer operative times in complex cases. Notably, these studies were based on non-robotic approaches and defined complexity solely according to anatomical criteria.

In contrast, our study identified higher rates of major postoperative complications, increased reintervention rates, and longer median hospital stays in patients undergoing complex segmentectomies. This discrepancy may be attributed to our broader definition of complexity, which does not rely on anatomical criteria but instead incorporates intraoperative factors such as prolonged operative time, conversion to open surgery, and unplanned changes in the extent of resection. By adopting this composite definition, we likely captured a wider range of technically demanding scenarios, providing a more accurate reflection of real-world surgical complexity. These findings highlight the importance of tailored perioperative strategies and careful case selection, particularly in the context of complex robotic segmentectomy.

Our study has several limitations. First, the sample size was small and derived from a single institution, which limits the generalizability of our findings. Although formal power calculations were not feasible, we believe that our sample size allows for meaningful interpretation of the identified associations. The definition of complex procedures based primarily on procedural duration may limit the generalizability of the exact cut-off, but similar sample sizes have been used in prior studies,^20^ providing context for interpretation of our results. Moreover, none of the associations reached conventional statistical significance (*P* < .05), and therefore the findings should be interpreted as exploratory. Nevertheless, they may provide valuable insights into potential factors influencing surgical complexity. In addition, although the regression model showed modest discrimination, it was intended to explore associations rather than to build a clinically applicable prognostic tool. External validation will be necessary for any future prognostic models based on these findings. Second, the score was developed using a cohort of patients operated on by 2 expert robotic surgeons. This approach aimed to minimize potential confounding related to variability in surgical technique, experience, intraoperative decision-making, and threshold for conversion. All procedures were performed after the initial learning curve for robotic anatomical resections had been overcome at our centre—approximately 6 months after the programme was initiated. Nonetheless, we cannot completely exclude the influence of a residual learning curve specific to robotic segmentectomy, which has been estimated at 21 cases according to Zhang et al.^29^

## CONCLUSION

This exploratory analysis identified preoperative factors potentially associated with increased surgical complexity in robotic segmentectomy based on patient characteristics and preoperative CT findings, rather than focusing solely on stapling technique, although this remains the most influential factor. Clinically relevant variables were retained in the analysis using a *P* < .10 threshold. Recognizing these factors may assist in preoperatively identifying appropriate candidates, contributing to improved surgical planning, resource allocation, and management of the learning curve. Complex cases were associated with increased major morbidity, reintervention rates, and longer hospital stay, highlighting the inherent risks of more demanding procedures and underscoring the need for tailored perioperative strategies in such cases.

## Supplementary Material

ivaf294_Supplementary_Data

## Data Availability

The data underlying this article will be shared on reasonable request to the corresponding author.
